# Bovine herpesvirus meningoencephalitis in the State of Tocantins, Brazil

**DOI:** 10.29374/2527-2179.bjvm004023

**Published:** 2024-01-30

**Authors:** Ilgner Aimar Bezerra Pinheiro, Bianca Pereira Dias, Jardel Martins Ferreira, Alessandro José Ferreira dos Santos, Sandro Estevan Moron, Gilzelle Maria da Luz Silva, Liana Bezerra Dias de Lima, Fabiano Mendes de Cordova

**Affiliations:** 1 Undergraduate in Veterinary Medicine, Liga Acadêmica Veterinária de Patologia, Universidade Federal do Norte do Tocantins, Araguaína, TO, Brazil; 2 Veterinarian, Núcleo de Estudos Avançados em Geoprocessamento e Estatística, Agência de Defesa Agropecuária do Estado do Tocantins, Palmas, TO, Brazil; 3 Biologist, Laboratório de Morfofisiologia e Bioquímica de Peixes Neotropicais, Universidade Federal do Norte do Tocantins, Araguaína, TO, Brazil; 4 Veterinarian, Laboratório de Patologia Experimental, Universidade Federal do Norte do Tocantins, Araguaína, TO, Brazil

**Keywords:** neuropathy, necrotizing meningoencephalitis, herpetic encephalitis, BoHV, cattle, neuropatia, meningoencefalite necrosante, encefalite herpética, BoHV, bovinos

## Abstract

Three outbreaks of herpesvirus meningoencephalitis in cattle have been reported in three municipalities in the northern region of the State of Tocantins, Brazil. In one outbreak, 41 predominantly young bovines were affected, with 2-3 deaths in some cases. The animals showed neurological signs of incoordination, blindness, and recumbency, with death occurring within approximately 4-5 d. At necropsy, hyperemia and leptomeningeal hemorrhages were observed in the brain. Histology revealed more intense lesions in the rostral portions of the brain, mainly affecting the frontoparietal cerebral cortex, with nonsuppurative encephalitis and meningitis, glial nodules, neuronophagia, and eosinophilic intranuclear inclusion bodies in the astrocytes and neurons. This study shows the presence of bovine herpesvirus in Tocantins, probably the highly neurotropic type 5 strain, and emphasizes its importance in the differential diagnosis of bovine neuropathies.

## Resumo

Três surtos de meningoencefalite por herpesvírus em bovinos são relatados em três municípios da região norte do Estado do Tocantins, Brasil. Num surto, 41 animais predominantemente jovens foram afetados, com 2-3 mortes nos outros casos. Os animais apresentaram sinais neurológicos de incoordenação, cegueira e decúbito, com a morte ocorrendo em aproximadamente 4 a 5 dias. Na necropsia foram observadas hiperemia e hemorragias leptomeníngeas no encéfalo. A histologia revelou lesões mais intensas nas porções rostrais do encéfalo, principalmente no córtex cerebral frontoparietal, com encefalite e meningite não supurativas, nódulos gliais, neuronofagia e corpúsculos de inclusão intranucleares eosinofílicos nos astrócitos e neurônios. Este estudo demonstra a presença do herpesvírus bovino no Tocantins, provavelmente a cepa tipo 5 altamente neurotrópica, e enfatiza sua importância no diagnóstico diferencial das neuropatias bovinas.

## Introduction

Bovine herpesvirus meningoencephalitis is a disease of the central nervous system (CNS) characterized by nonsuppurative inflammation and necrosis, which is more pronounced in the frontal lobes of the cerebral cortex (Rissi et al., 2006). The disease is mainly caused by a highly neurotropic strain of bovine herpesvirus type 5 (BoHV-5), which is a double-stranded and enveloped deoxyribonucleic acid (DNA) virus belonging to the Herpesviridae family, Alphaherpesvirinae subfamily, and *Varicellovirus* genus (Gomes et al., 2002; Rissi et al., 2008). However, the disease can also involve herpesvirus type 1 (BoHV-1), a genetically and antigenically similar strain (Del Médico Zajac et al., 2010; Delhon et al., 2003) that is usually associated with infectious bovine rhinotracheitis (IBR), infectious pustular vulvovaginitis, infectious pustular balanoposthitis, conjunctivitis, abortion, and systemic disease of newborn calves (Muylkens et al., 2007; Silva et al., 2007a).

BoHV-5 meningoencephalitis is characterized by low morbidity and high lethality (Rissi et al., 2007). It usually affects young cattle up to 18 months of age (Blume et al., 2018; Bulach & Studdert, 1990), although older animals can also be affected (Maidana et al., 2011). The disease is associated with various clinical signs, including tremors; circling; incoordination; depression; loss of visual, auditory, or skin reflexes; blindness; mandibular trismus; bruxism; nystagmus; opisthotonos; paresis; permanent recumbency; pedaling movements; tetany; convulsions; and death in a clinical course of 4-15 d (Aquino Neto et al., 2009; Bagust & Clark, 1972; Blume et al., 2018; Cagnini et al., 2017; Carrillo et al., 1983; Meyer et al., 2001; Silva et al., 2007.

Cases of disease associated with BoHV-5 have a higher prevalence in Australia, Brazil, and Argentina (Rissi et al., 2007) and few or rare cases in the US and Europe (Del Médico Zajac et al., 2010). In Brazil, the disease has been reported in the States of Goiás (Blume et al., 2018; Freitas Neto et al., 2010; Paula et al., 2005), Mato Grosso (Arruda et al., 2010; Claus et al., 2007; Colodel et al., 2002), Mato Grosso do Sul (Claus et al., 2007; Gomes et al., 2002; Salvador et al., 1998; Silva et al., 2007a), Pará (Riet-Correa et al., 2006), Paraíba, Rio Grande do Norte (Galiza et al., 2010), Paraná (Claus et al., 2007; Lunardi et al., 2009; Massitel et al., 2016), Pernambuco (Oliveira et al., 2014), Rio de Janeiro, Minas Gerais (Aquino Neto et al., 2009; Claus et al., 2007; Gomes et al., 2002; Oliveira et al., 2016), Rio Grande do Sul (Elias et al., 2004; Riet-Correa et al., 1989; Rissi & Barros, 2013; Rissi et al., 2006, 2007, 2008; Sanches et al., 2000; Silva et al., 2007b; Weiblen et al., 1989), and São Paulo (Claus et al., 2007; Ferrari et al., 2007; Gomes et al., 2002; Salvador et al., 1998). In some regions of Brazil, meningoencephalitis caused by BoHV-5 has become one of the most important diseases of cattle (Blume et al., 2018; Colodel et al., 2002; Sanches et al., 2000), observed in the form of outbreaks or individual cases, with low morbidity and mortality, high lethality, and no seasonal pattern (Barros et al., 2006; Colodel et al., 2002; Elias et al., 2004; Freitas Neto et al., 2010; Lunardi et al., 2009; Paula et al., 2005; Riet-Correa et al., 2006; Rissi et al., 2006; Salvador et al., 1998).

Due to the importance of the disease in several regions of Brazil and the lack of information related to the neurological form of BoHV infection in the State of Tocantins, we aimed to describe the epidemiological and clinicopathological findings of three natural cases of herpetic meningoencephalitis diagnosed in this state.

## Case description

Data on the epidemiology and clinical signs were obtained from veterinarians on the properties or from the Official Veterinary Service of the Agência de Defesa Agropecuária of the State of Tocantins (ADAPEC-TO), Araguaína, TO, Brazil, who provided services on the properties of the three affected cases. In the first case, a bovine (animal 1) in excellent physical condition (body score 4/5) was necropsied in February 2022. The brain, spinal cord, and fragments of thoracic and abdominal organs were collected and fixed in 10% formaldehyde. In the second case, a bovine (animal 2) was necropsied in April 2022, and the brain and spinal cord collected and preserved in 10% formaldehyde. The third case (animal 3), attended to due to neurological syndromes in October 2013. A necropsy was carried out and CNS samples were collected. After fixing, the CNS fragments from animals 1 and 2, serial cross-sections of fragments of the cerebral cortex, basal ganglia, thalamus, midbrain, colliculi, cerebellar peduncles, cerebellum, pons, medulla oblongata, and cervical spinal cord were performed. Samples were routinely processed and stained with hematoxylin and eosin for histopathological examination.

**Figure 1:**
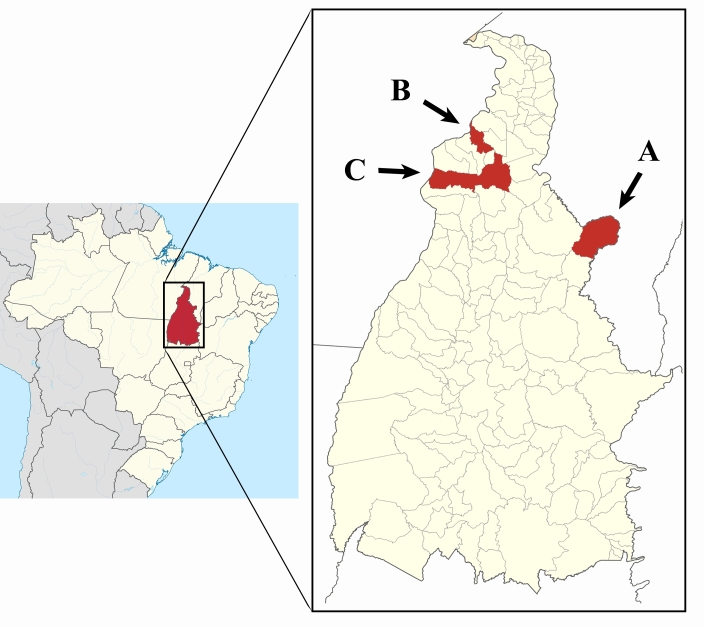
Location of municipalities in the State of Tocantins, Brazil, with cases of bovine herpesvirus meningoencephalitis. (A) Campos Lindos, (B) Araguanã and (C) Araguaína. Images adapted from Wikimedia Commons (Wikipédia, 2023). Licenses for Map of Brazil CC-BY-SA-3.0 (TUBS - Wikimedia Commons, 2013) and Map of the State of Tocantins CC-BY-2.5 (Milenioscuro - Wikimedia Commons, 2021).

The disease was observed in the municipalities of Campos Lindos (case 1, February 2022), Araguanã (case 2, April 2022), and Araguaína (case 3, October 2013), TO, Brazil, which are all located in the northern region of the state (Figure 1). Aberdeen Angus animals (case 1) and crossbreeds (cases 2 and 3) raised semi-intensively and extensively, respectively were also affected. Case 1 developed as an outbreak, with 41 cattle deaths on the property, predominantly young cattle (39 animals) aged 3-6 months old and two 2-year-old cows. Animal deaths began in December 2021, but only in February 2022 was a bovine (animal 1) sent for necropsy. The animals showed signs of a progressive neurological condition, with difficulty keeping themselves upright, locomotor difficulties, apparent blindness, entry into a comatose state, and permanent lateral decubitus, culminating in death. The clinical course of the disease ranged 5-8 d. A veterinarian was asked to evaluate the farm, and raised suspicion of botulism, with a water source as a possible carrier of botulinum toxin. Owners instituted a vaccination program for clostridiosis and rabies in January 2022 and observed a subsequent reduction and cessation of new cases by the end of February 2022.

Case 2 affected two animals in the herd, both 3-month-old calves, but CNS samples from only one cow (animal 2) were examined. The calf presented with the onset of a neurological condition with motor incoordination and proprioceptive loss of the right pelvic limb and, development of flaccid paralysis of the pelvic limbs within 24 h. Subsequently, the animal went into permanent recumbency and progressed to death four days after the onset of clinical signs.

Case 3 occurred during an investigation of neurological syndromes by the state's official veterinary service. Three cattle on the farm, all approximately 12-months-old, were affected. The animals showed similar clinical signs, with an alert mental state, motor in-coordination with mild ataxia, walking in circles, bruxism, hypotonia of the tongue, preserved pupillary reflexes, evolution of the reduction of reflexes, positioning in lateral decubitus, opisthotonos, and death. One of the animals was necropsied, and CNS samples were sent to a reference laboratory and tested negative for rabies and positive for herpetic meningoencephalitis.

**Figure 2:**
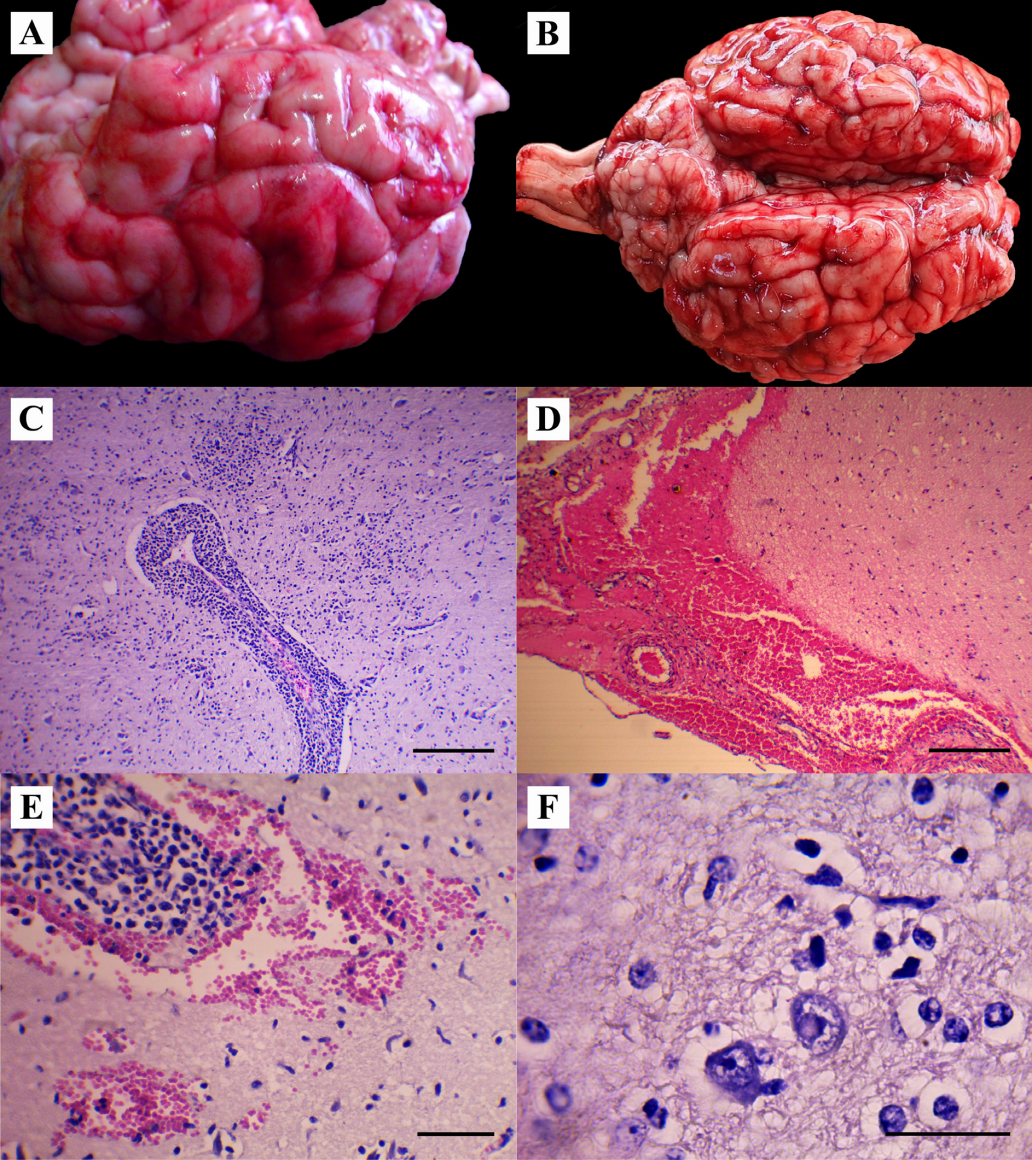
Anatomopathological evaluation of bovine herpesvirus meningoencephalitis in the State of Tocantins. Macroscopic aspect of the brains of animals 1 (A) and 2 (B), showing hyperemia and foci of hemorrhage in the leptomeninges. (C) Intense lymphocytic inflammatory infiltrate with perivascular cuff formation, glial nodules, and neuronal necrosis in the frontoparietal cerebral cortex (animal 1; scale bar 100 μm). Areas of leptomeningeal (D, animal 2; scale bar 100 μm) and parenchymal (E, animal 1; scale bar 50 μm) hemorrhages in the cerebral cortex. (F) Eosinophilic intranuclear inclusion body in a neuron (animal 1; scale bar 20 μm).

Macroscopically, the brains of animals 1 and 2 showed hyperemia and scattered hemorrhagic foci in the leptomeninges (Figure 2A), which were more prominent in bovine 2 (Figure 2B), which also showed diffuse and bilateral pulmonary congestion. Microscopically, the alterations in animals 1 and 2 were similar, affecting the frontal (more intensely), temporal, parietal, and occipital cerebral cortices (less intensely), mesencephalic region, pons, and bulb (discretely). Diffuse and accentuated leptomeningeal lymphocytic infiltrates (Figure 2C) and foci of leptomeningeal and parenchymal hemorrhages were also observed in these areas (Figure 2D and 2E), with cortical intercellular edema and status spongiosus in the white matter, which was significant in animal 2. The neuropile showed multifocal areas of glial nodule formation and neuronophagia, where neurons exhibited shrunken, hypereosinophilic cytoplasm and pyknotic nuclei. Multifocally throughout the neuropil and adjacent neuroparenchyma, the blood vessels showed prominent endothelial cells exhibiting perivascular spaces with cuffs (up to eight cells thick) of lymphocytes, few plasma cells, and rare neutrophils (Figure 2C), which extended to the leptomeningeal vessels. Few neurons and occasional astrocytes contained eosinophilic intranuclear viral inclusions (Figure 2F), sometimes pushing chromatin peripherally. No lesions were observed in the cerebellum or cervical spinal cord.

## Discussion

BoHV meningoencephalitis was diagnosed based on epidemiological, clinical, and characteristic histological findings, with predominant involvement of young animals, nonsuppurative encephalitis and meningitis, and intranuclear corpuscle inclusions in astrocytes and neurons (Rissi & Lemos, 2023). Clinical signs and necropsy findings such as those reported in this study are important for diagnosing BoHV meningoencephalitis. However, other diseases that affect the CNS of cattle with neurological clinical signs, such as polioencephalomalacia and rabies, should be considered as differential diagnosis.

In case 1, according to the information obtained from the farm veterinarian, problems related to neurological disorders were occurring 3 months earlier with animals with similar neurological signs, which began with motor incoordination and evolved to decubitus and death. Interestingly, the involvement of animals on the farm was reduced after the peak of the disease (in January 2022) until it disappeared in February when we received a bovine for necropsy. Coincidentally, the reduction and disappearance of the disease in this outbreak occurred after vaccination of the herd for clostridiosis and rabies, which could suggest the circulation of the etiological agents of these diseases in the region. However, herpetic meningoencephalitis was diagnosed based on histopathological findings (corpuscles of inclusion). In addition, no records of rabies were found in the Campos Lindos region during the study period related to the case (Tocantins, 2022). Thus, the relationship between vaccination and the disappearance of the disease on the farm has been characterized as a coincidence because herpetic meningoencephalitis has self-limiting behavior, with the virus entering latency, and can be reactivated in stressful situations, such as animal management, weaning, or transportation (Del Médico Zajac et al., 2010; Meyer et al., 2001; Perez et al., 2002).

In case 2, two calves had a neurological manifestation; however, only one was examined. The clinical profile began with low spinal cord syndrome, with symptoms of pelvic limb incoordination, an uncommon presentation (Aquino Neto et al., 2009), evolving to loss of mobility, decubitus, and death. Notably, according to information from ADAPEC-TO veterinarians, this farm in the municipality of Araguanã had received animals from the region of Campos Lindos (where case 1 occurred) a few weeks before the episode of the disease on the property. This case may exemplify a situation of virus dissemination between two noncontiguous and relatively distant regions (approximately 340 km) in the state.

In case 3, the affected animals were recently weaned and transported from the southern region of Tocantins State to the destination farm in the municipality of Araguaína, located in the northern region of the same state. Upon arrival, the animals were handled in the corral and branded with hot iron, which is a stressful situation that corroborates the reports described in scientific literature (Barros et al., 2006; Rissi et al., 2007). On clinical examination, the animals showed an alert mental state, motor in-coordination with mild ataxia, walking in circles, bruxism, and tongue hypotonia, but preserved pupillary reflexes and pairs of cranial nerves and upper and lower motor neurons. Subsequently, these reflexes decreased, and the animal remained in the lateral decubitus position, showed opisthotonos, and eventually died. These clinical signs were consistent with those described by Rissi and Lemos (Rissi & Lemos, 2023) in animals infected with BoHV-5.

Despite the epidemiological, clinical, and anatomopathological characteristics, stating that the cases studied were due to a type 5 BoHV infection is impossible. No specific tests were performed to determine the viral strains. However, the type 5 virus is probably involved because of its highly neurotropic features (Rissi & Lemos, 2023). Although BoHV-1 and BoHV-5 variants are genetically and antigenically related, their neuroinvasion and neurovirulence capabilities differ (Del Médico Zajac et al., 2010). BoHV-1 usually does not invade beyond the first-order neuron located in the trigeminal ganglion, where the latent infection is established, while BoHV-5 spreads to different regions of the brain (Carrillo et al., 1983; Perez et al., 2002; Vogel et al., 2003). Although not characteristic, cases of BoHV-1 encephalitis have also been reported (Roels et al., 2000; Silva et al., 2007.

The disease has not yet been described in the Brazilian state of Tocantins. Although it occurs in several other states in Brazil (Aquino Neto et al., 2009; Arruda et al., 2010; Blume et al., 2018; Claus et al., 2007; Colodel et al., 2002; Elias et al., 2004; Ferrari et al., 2007; Freitas Neto et al., 2010; Galiza et al., 2010; Gomes et al., 2002; Lunardi et al., 2009; Massitel et al., 2016; Oliveira et al., 2014, 2016; Paula et al., 2005; Riet-Correa et al., 1989, 2006; Rissi & Barros, 2013; Rissi et al., 2006, 2007, 2008; Salvador et al., 1998; Sanches et al., 2000; Silva et al., 2007b; Weiblen et al., 1989), particularly in the neighboring States of Pará (Riet-Correa et al., 2006), Goiás (Blume et al., 2018; Freitas Neto et al., 2010; Paula et al., 2005), and Mato Grosso (Arruda et al., 2010; Claus et al., 2007; Colodel et al., 2002), where it is considered one of the main cattle diseases (Blume et al., 2018; Colodel et al., 2002), BoHV is likely circulating throughout the state of Tocantins. One study demonstrated the presence of both BoHV-1 and BoHV-5 in the extreme south of Tocantins state, in the municipalities of Alvorada, Cariri, Itaporã, Sucupira, and Aliança (Silva, 2011). Despite the low prevalence and morbidity of the disease (Rissi & Lemos, 2023), it is likely to be more frequent, and many outbreaks are not diagnosed, as they are isolated cases in extensive livestock farming or farms which are distant from diagnostic laboratories. Moreover, definitive diagnoses were not achieved in many cases attended for neurological syndromes in cattle by Official Veterinary Services. With the exclusion of rabies and spongiform encephalopathy (for corresponding epidemiological cattle), biological samples were not routinely subjected to other examinations.

## Conclusions

This is the first report of herpetic meningoencephalitis in the State of Tocantins and, although little is known about the occurrence of the disease, the scarcity of reports of this nature highlights the need for further studies on the distribution of BoHV in different regions of the state. In this respect, although herpetic meningoencephalitis is apparently sporadic, the diagnosis of the disease is important, particularly in the differential diagnosis of rabies, as well as other diseases related to similar nerve symptomatology. Thus, establishing a diagnostic routine that allows the determination of diseases that affect the nervous system of cattle in Tocantins is necessary.
